# Efficient Modeling of Quantum Dynamics of Charge Carriers in Materials Using Short Nonequilibrium Molecular Dynamics

**DOI:** 10.1021/acs.jpclett.3c02187

**Published:** 2023-09-08

**Authors:** Bipeng Wang, Yifan Wu, Dongyu Liu, Andrey S. Vasenko, David Casanova, Oleg V. Prezhdo

**Affiliations:** † Department of Chemical Engineering, University of Southern California, Los Angeles, California 90089, United States; ‡ Department of Chemistry, University of Southern California, Los Angeles, California 90089, United States; § HSE University, 101000 Moscow, Russia; ∥ Donostia International Physics Center (DIPC), 20018 San Sebastián-Donostia, Euskadi, Spain; ⊥ IKERBASQUE, Basque Foundation for Science, 48009 Bilbao, Euskadi, Spain

## Abstract

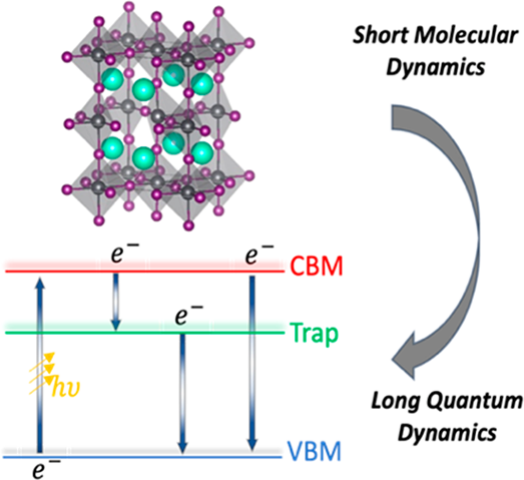

Nonadiabatic molecular dynamics provides essential insights into excited-state processes, but it is computationally intense and simplifications are needed. The classical path approximation provides critical savings. Still, long heating and equilibration steps are required. We demonstrate that practical results can be obtained with short, partially equilibrated ab initio trajectories. Once the system’s structure is adequate and essential fluctuations are sampled, the nonadiabatic Hamiltonian can be constructed. Local structures require only 1–2 ps trajectories, as demonstrated with point defects in metal halide perovskites. Short trajectories represent anharmonic motions common in defective structures, an essential improvement over the harmonic approximation around the optimized geometry. Glassy systems, such as grain boundaries, require different simulation protocols, e.g., involving machine learning force fields. 10-fold shorter trajectories generate 10–20% time scale errors, which are acceptable, given experimental uncertainties and other approximations. The practical NAMD protocol enables fast screening of excited-state dynamics for rapid exploration of new materials.

Nonadiabatic (NA) molecular dynamics (MD) has proven to be a key tool in the investigation and understanding of far-from-equilibrium processes in molecular and nanoscale systems.^1−10^ This is particularly important when dealing with complex, multicomponent systems that exhibit a rich variety of dynamical phenomena. NAMD simulations are computationally more demanding than calculations of electronic ground- and excited-state properties of systems in optimized geometries, or adiabatic MD, or rates of transitions between electronic states. NAMD simulations include all these components plus solutions of the time-dependent Schrödinger equation and ensuing surface-hopping algorithms. Transition rates in NAMD are explicitly configuration- and time-dependent and avoid the weak coupling and harmonic phonon assumptions typically used in rate expressions, such as Fermi’s golden rule. A full-scale NAMD simulation requires sampling of hundreds of excited-state MD trajectories and evaluation of NA coupling (NAC) matrix elements on the fly, making the simulation very demanding.

Often, molecules that are stable in the ground electronic state can undergo a chemical reaction, e.g., bond breaking, after excitation, with the process strongly depending on the excited state(s) involved. In comparison, the electronic excitation of a material usually has little influence on its thermal atomic fluctuations. Or if it does, as in a photoinduced phase transition,^11^ the material rapidly relaxes through a dense manifold of electronic states, and the excitation can be modeled as a single particle transition across the bandgap. Density functional theory (DFT) typically provides an efficient and accurate description of a material’s electronic properties, and excitation of one or a few electrons has a small effect on the electron density that enters the DFT functional. These features justify the use of the classical path approximation (CPA), in which multiple and computationally expensive excited-state trajectories are replaced by a single ground-state (GS) trajectory used to sample the NA Hamiltonian.^12^ Indeed, ab initio NAMD under the CPA has proven to be a reliable tool for analyzing excited-state dynamics in a broad range of materials.^13−21^

On one hand, the structural response of a material to an electronic excitation is generally simpler than that of a molecule. On the other hand, a molecule is chemically unique with a limited number of structural conformers, while there exist many versions of the same material, varying in defects, dopants, stoichiometry, thermodynamics phase, surfaces and their passivation, grain boundaries, etc. There is a need to investigate multiple structures associated with a particular material in order to accurately assess a material’s properties. For example, there can exist a dozen or more different types of point defects in a material, and such defects can have a strong influence on the excited-state dynamics. Combinations of point defects, grain boundaries, dopants, and so on create a huge number of structures. The need to accurately represent excited-state evolutions in molecules is replaced in materials by the need to sample many structures, creating a new computational challenge. Further challenges arise from the large size of materials simulations. For example, modeling of point defects requires supercells composed of multiple unit cells, needed to eliminate spurious interactions between periodic images of defects.

The quest for efficient MD approaches has resulted in several methodologies. Coarse-grained MD cuts down the computational time by representing groups of atoms as single entities.^22^ However, a coarse-grained representation changes the meaning of the MD time arrow and makes problematic the evaluation of the NAC that is a time-derivative of the electronic wave function. Coarse graining also limits our understanding of electronic properties, for example, of atomic point defects. The hybrid quantum mechanical/molecular mechanical (QM/MM) approach provides another direction to manage complex calculations.^23,24^ It employs detailed quantum mechanical methods on critical system areas while using simpler molecular mechanical methods elsewhere. This approach makes it possible to study large, complex structures. QM/MM NAMD simulations are particularly useful if the system can be separated naturally into weakly interaction parts, such as a molecular chromophore in a solution or a protein matrix, or a solid/liquid interface.^25,26^ Treatment of the interaction between the QM and MM regions is the critical aspect of QM/MM models. For example, it is not trivial to surround a QM point defect in a material with an MM bulk. Various embedding methods have been developed for this purpose.^27,28^ Recent advances in machine learning (ML) models of electronic Hamiltonians provide another avenue for increasing computational efficiency.^29−31^

In addition to the large size and multiple structures possible in a material, one must consider simulation time. Some materials are stiff and undergo only small-scale harmonic motions around equilibrium geometries. Other systems are softer and can exhibit very anharmonic motions on long time scales. The time scale challenge can be addressed by ML models of force fields^32^ and NAC.^33,34^ However, construction of such models is conditional on thorough training based on large data sets that require thousands of ab initio calculations. Therefore, minimizing the cost of such simulations is an important practical goal.

In this work, we analyze the computational cost required to obtain practically useful NAMD results for rapid screening of new materials and exploration of their excited-state dynamics time scales. We show that MD trajectories that barely achieve equilibration and sample only a few periods of characteristic oscillations of material structure are sufficient to obtain NAMD results within 10–20% accuracy of a thorough NAMD simulation. Such accuracy is sufficient given the various theoretical approximations and experimental uncertainties involved. Modern material structures contain defects, dopants, nonstoichiometric compositions, etc. and exhibit anharmonic motions, and therefore, using short MD trajectories in place of the harmonic approximation around the optimized geometry is essential and can lead to qualitatively different conclusions. The demonstrated NAMD simulation protocol expedites computational screening of excited-state dynamics across a multitude of materials without compromising the reliability of the results.

To establish the minimal computational time needed to obtain useful NAMD results, we consider metal halide perovskites (MHPs), which are at the focus of many experimental and theoretical studies and which present significant computational challenges because of the many coupled processes and multiple time scales involved.^35−40^ MHPs have garnered significant attention over the past decade due to their outstanding optoelectronic properties, making them promising candidates for use in next-generation solar cells, light-emitting diodes, and other electronic devices.^41−43^ With their unique blend of low-cost fabrication, tunable band gaps, high charge carrier mobility, and exceptional light absorption capabilities, MHPs are modernizing photovoltaics and optoelectronics. The advantageous properties stem from the MHP unique structure, in which metal cations and halide anions form an inorganic framework that supports efficient charge transport and sandwiches an organic or inorganic cation. The cation does not contribute directly to the electronic properties at the most important energy range but influences geometric structure through local steric interactions and charge carriers via long-range electrostatic forces. MHPs exhibit extremely favorable properties of defects^44^ limiting carrier losses and a distinctive interplay of solid-state and molecular properties. Despite their potential, MHPs are not without problems. They exhibit complex photophysical behavior influenced by defects and structural dynamics, leading to stability issues and current–voltage hysteresis.^45−47^ Effective utilization and optimization of MHPs require understanding the structural and electronic dynamics achievable by NAMD.

The computations were carried out using density functional theory (DFT) as implemented in the Vienna Ab initio Simulation Package (VASP).^48−50^ The Perdew–Burke–Ernzerhof (PBE) density functional^51^ was used in conjunction with projector augmented wave (PAW) pseudopotentials.^52,53^ The plane wave basis cutoff energy was set at 450 eV, and the energy convergence threshold was 10^–7^ eV. Three different MHP systems were considered: CsPbI_3_ with a replacement defect, Cs replaced by I (Cs_I_); CsPbI_3_ with an iodine interstitial defect (I_i_); and CsPbI_3_ with an iodine vacancy defect (I_v_), Figure 1. Each tetragonal defective CsPbI_3_ model was built using a 2 × 2 × 2 supercell. The VESTA software package^54^ was employed for visualizing the structures.

**Figure 1:**
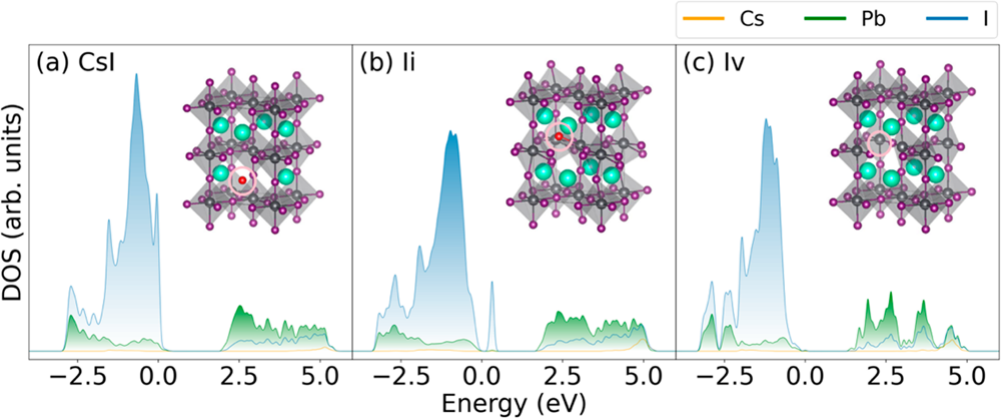
Atomic-projected DOS for tetragonal CsPbI_3_ with different defects: (a) Cs replaced by I (Cs_I_), (b) I interstitial (I_i_), and (c) I vacancy (I_v_). Zero energy is set to the VBM. The insets show the optimized structures. The defective atom is circled and marked red. Only I_i_ creates a midgap state in the optimized structure.

The goal of the present work is to evaluate the minimal amount of ab initio simulations required to obtain results that can provide explanation and guidance for experimental work. Typically, ab initio NAMD simulations are performed using multiple stages. They include geometry optimization, heating, and adiabatic MD to sample a canonical distribution of initial conditions for NAMD. Under the CPA, the adiabatic MD trajectory is also used to calculate the NA Hamiltonian. These parts are the most time-consuming, since the subsequent solving of the time-dependent Schrödinger equation and sampling multiple realizations of surface hopping (SH) along the precomputed trajectory are typically fast, unless the electronic state basis is very large.^55^ Thus, the main goal is to reduce the number of optimization, heating, and adiabatic MD parts. In particular, we show that good results can be obtained just from the heating trajectory, skipping adiabatic MD altogether.

We start with the experimental crystal structure of pristine CsPbI_3_, maintain the lattice constants and shape, and optimize the geometry of pristine CsPbI_3_ with DFT. Then, we introduce the point defects and optimize the geometries. Although one can skip geometry optimization and start heating/equilibration immediately, the introduction of defects can create a strong perturbation to the pristine lattice, and it is safer to optimize the geometry first. Then, we heat the structures for 2 ps to reach 300 K. The heating is accomplished by velocity rescaling with a 1 fs atomic time step. The final 1 ps of the 2 ps heating trajectories is used to calculate the NA Hamiltonian, iterate it multiple times, and perform NAMD. For brevity, we call this the ShortMD-NAMD protocol. We compare this minimalistic scheme to conventional NAMD calculations. Here, we maintain the systems at 300 K for an additional 4 ps to ensure system equilibration. Then, we perform a 10 ps MD simulation in the microcanonical ensembles. The last 7 ps of the trajectories is used to calculate the NA Hamiltonian, iterate it, and perform NAMD. We call this protocol LongMD-NAMD.

NACs are computed numerically through the evaluation of wave function overlap at successive MD timesteps,^12^ adapted for the PAW method.^56,57^ The NAMD simulations are performed using the decoherence-induced surface hopping (DISH) methodology,^58,59^ as implemented in the Pyxaid software.^12,59^ The pure-dephasing times are estimated via the second-order cumulant approximation of the optical response theory.^60^ The error bars of the Gaussian fits are small, 10^–4^–10^–5^. A total of 100 initial geometries are sampled, and 1000 realizations of the stochastic DISH process are generated for each initial geometry.

Figure 1 presents the optimized structures of CsPbI_3_ featuring the Cs_I_, I_i_, and I_v_ defects, together with their respective projected density of states (DOS). CsPbI_3_ with the Cs_I_ and I_v_ defects display a direct bandgap between the valence band maximum (VBM) and the conduction band minimum (CBM), with the respective 0 K gaps of 1.79 and 1.61 eV. Introduction of the I_i_ defect creates a trap state located 0.30 eV above the VBM. The state contains one electron and is half-filled. Therefore, it can trap both electron and hole. In all cases, the VBM is mainly associated with I atoms, whereas the CBM is primarily attributed to Pb atoms.

Figure 2 illustrates the evolution of the VBM, trap, and CBM electronic energy levels in the three systems. The 6 ps heating phase is separated from the 10 ps microcanonical ensemble calculation by a dotted line. The shaded regions illustrate the parts used to calculate the NA Hamiltonian in the ShortMD-NAMD and LongMD-NAMD simulation protocols.

**Figure 2:**
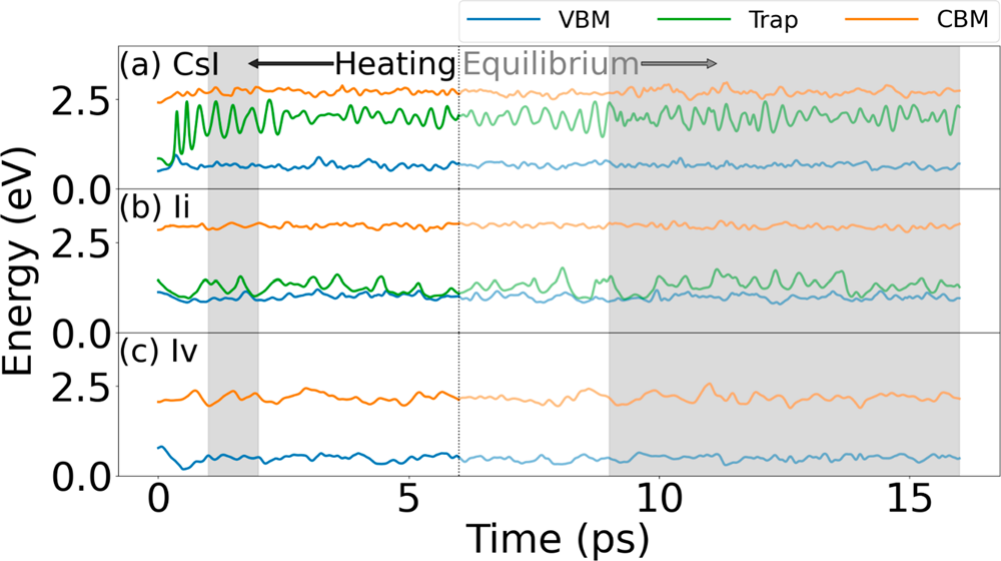
16 ps evolution of the VBM, trap, and CBM energy levels of CsPbI_3_ with (a) Cs_I_, (b) I_i_, and (c) I_v_ defects. Following a typical protocol, each system is heated to 300 K for 6 ps in the NVT calculation. Then, as indicated by the vertical dotted line, a 10 ps production run is performed in the NVE ensemble. The two shadow regions correspond to the two simulation scenarios. Typically, the last 7 ps are used to sample the NA Hamiltonian and perform NAMD. In the tested approach, only the 1 to 2 ps part of the NVT trajectory is used instead. The proposed approach provides an 8-fold computational saving since it requires a 2 ps simulation instead of a 16 ps simulation. Note that the energy of the Cs_I_ defect state moves from the VBM in the optimized geometry to the CBM at ambient temperature, and this process must be captured prior to sampling the NA Hamiltonian.

The Cs_I_ system demonstrates an important phenomenon that limits the minimal MD duration needed to obtain good NAMD results. The Cs_I_ defect state, located at the VBM in the optimized structure (Figure 1a), rises high into the bandgap, approaching the CBM (Figure 2a). The structural change gives rise to such a significant change in the electronic properties and must be properly accounted for.

The I_i_ defect demonstrates another important feature. The I_i_ state is located 0.3 eV above the VBM at 0 K (Figure 1b). In comparison, at room temperature, the state fluctuates by several tenths of an electronvolt, often approaching the VBM. At instances when the VBM–trap energy gap is transiently bridged, the trapped hole can escape into the valence band. Such large fluctuations of energy levels are typical of most defects in MHPs, because MHPs are soft.^61^ The I_i_ defect is deep in the optimized structure; compare the 300 meV separation of the I_i_ defect level from the VBM (Figure 1b) to *k*_B_*T* = 25 meV at room temperature. However, the same level becomes shallow transiently at ambient conditions, rationalizing why MHP defects are considered to be shallow.^62−64^ The thermal fluctuation of the defect levels should be taken into account in order to achieve meaningful NAMD results.

The CBM and VBM energy levels in CsPbI_3_ with the I_v_ defect fluctuate trivially during the whole ab initio simulation, and no levels associated with the I_v_ species enter the bandgap (Figure 2c). However, on a much longer time scale on the order of 50–100 ps, deep midgap levels can appear transiently for the halide vacancy defects.^36,37,65,66^ Such a phenomenon requires special consideration, and it is hard to study by the direct ab initio methodology. Similarly, defect diffusion and its influence on charge carrier dynamics has to be treated by other means, such as transition path sampling.^47^

Considering typical properties of point defects, we observe that 1 ps is sufficient to allow for structural rearrangements and fluctuations that influence defect levels at ambient temperature, suggesting that the NA Hamiltonian can be obtained immediately following the 1 ps heating step, avoiding the need for the conventional 10 ps or longer ab initio MD calculations. Such a shortcut can create significant computational savings if one aims to study dozens of point defects, their combinations, point defects at grain boundaries and surfaces, etc.

The hypothesis presented above requires a quantitative analysis. The detailed data are provided in Tables 1 and S1–S3. In addition to the mean values of the energy gaps and mean absolute values of the NAC shown in Table 1, Tables S1–S3 report the corresponding root-mean-square value, which characterizes gap and NAC fluctuations. Consider the Cs_I_ defect. The mean VBM-trap, trap-CBM, and VBM-CBM energy gaps obtained from the short, partially equilibrated 1 ps NVT simulations are 1.24, 0.82, and 2.06 eV (left shaded region in Figure 2). The corresponding values from the long, fully equilibrated 7 ps NVE simulations are 1.33, 0.70, and 2.04 eV (right shaded region in Figure 2). The agreement is reasonable. The error in the VBM-CBM gap is 2%, while the errors for the gaps involving the trap state are 10–15%. Seemingly large, a 15% error is much smaller than experimental uncertainties in charge carrier lifetimes and time scale calculation errors stemming from various approximations used in NAMD and real-time time-dependent DFT.^10^

**Table 1:** Canonically Averaged Energy Gap, Absolute NAC, Recombination Time, and Pure-Dephasing Time for Short (1 ps NVT) and Long (7 ps NVE) Sampling in CsPbI_3_ with the Cs_I_ Defect

	Gap (eV)			Abs NAC (meV)		
CsI	VBM-Trap	Trap-CBM	VBM-CBM	VBM-Trap	Trap-CBM	VBM-CBM
1 ps NVT	1.24	0.82	2.06	0.29	1.48	0.30
7 ps NVE	1.33	0.70	2.04	0.24	0.90	0.27

		Pure-Dephasing Time (fs)		
	Recombination Time (ns)	VBM-Trap	VBM-CBM	Trap-CBM
1 ps NVT	54	2.50	7.83	2.50
7 ps NVE	41	3.76	5.96	3.40

Figure 3 shows the NACs for VBM-trap, trap-CBM, and VBM-CBM transitions in CsPbI_3_ with the Cs_I_ defect, corresponding to the shaded portions in Figure 2a. Generally, one expects structural changes to have a stronger influence on NAC than the energy. The short NACs demonstrate several oscillation cycles, capturing high and low peaks. The NAC magnitudes are comparable between the long and short trajectories (Table 1). The biggest difference is seen for the trap–CBM transition, with the average absolute NAC values of 1.48 and 0.90 meV for the short and long MD, respectively. This variation can be attributed to incomplete equilibration within the initial 2 ps, which results in larger fluctuations in the trap-state energy. The trap-CBM NAC exhibits a transient large value of nearly 10 meV at around 12 ps in the long MD simulation (Figure 3b). Such large values are absent in the short MD. The NAC is inversely proportional to the gap between states,^12,67^ and the trap state moves close to the CBM at around 12 ps in the Cs_I_ defect system (Figure 2a). Generally, high NAC peaks can accelerate electron–hole recombination. However, the high NAC value appears transiently, and its influence on the electron–hole recombination should not be very significant because the bottleneck is the transition between the trap state and the VBM.

**Figure 3:**
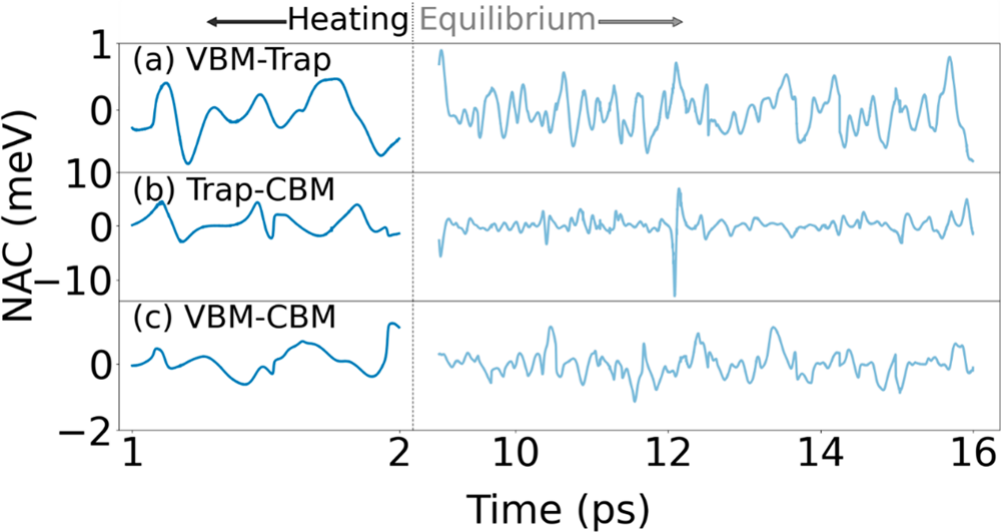
NAC of CsPbI_3_ with the Cs_I_ for the (a) VBM-trap, (b) trap-CBM, and (c) VBM–CBM transitions. Shown are only the regions selected to test the electron–hole recombination (gray areas in Figure 2). The heating and equilibrium simulations are divided by the vertical dotted line. NAC values of the I_i_ and I_v_ defects are shown in the Supporting Information. The 1 ps heating region provides a reasonable representation of the NAC in the 7 ps equilibrium region.

Figure 4 displays evolutions of the GS populations, describing GS recovery due to recombination of electrons and holes starting in the CBM and VBM, obtained by the NAMD simulations on CsPbI_3_ featuring the three point defects. Given that iodine possesses an odd number of electrons, the I_i_ trap state can function as both an electron (e) and hole (h) trap, with the two scenarios considered separately. For the Cs_I_ and I_i_ h-trap defects, the GS populations reach around 0.2 within 10 ns, corresponding to the recombination time of around 50 ns, as shown in Tables 1 and S1–S3. The GS population rises above 0.3 within 10 ns for the I_i_ e-trap and I_v_ defect simulations, indicating a faster charge recombination. In all instances, the results of the ShortMD-NAMD method are comparable to those of the LongMD-NAMD method, even though the former is based on partially equilibrated systems. The charge recombination times obtained with the ShortMD-NAMD method for Cs_I_, I_i_ e-trap, I_i_ h-trap, and I_v_ are 54, 24, 46, and 31 ns, respectively. The corresponding LongMD-NAMD values are 41, 46, 52, and 25 ns (Tables S1–S3). The differences are 10–20%, which is sufficient to obtain useful insights and to analyze experiments. The charge recombination time remains similar across all systems, regardless of the presence or absence of midgap trap states, reinforcing the notion that defects are typically harmless in lead halide perovskties.^61−63^ Overall, the ShortMD-NAMD method offers a reasonable approximation for modeling charge trapping and recombination in MHPs featuring common defects, reducing the computational effort by nearly 1 order of magnitude compared to the commonly used NAMD simulation protocol.

**Figure 4:**
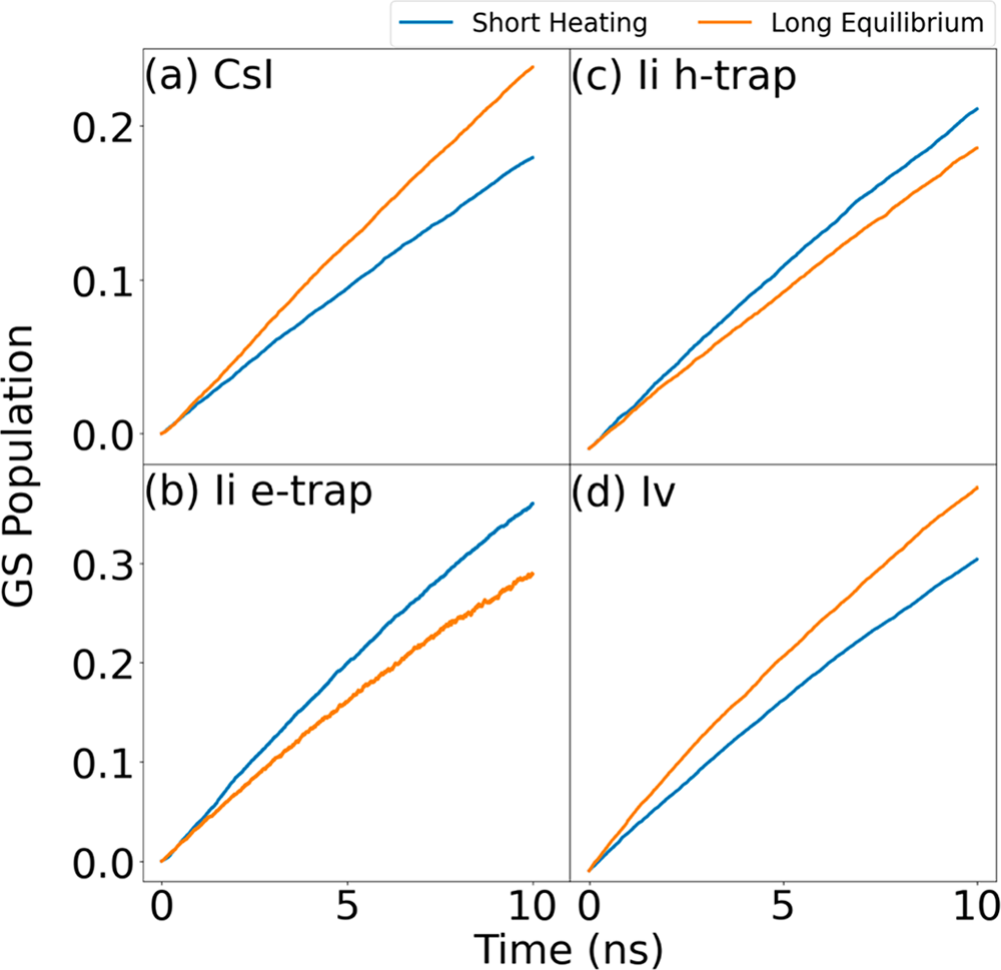
Evolution of the ground-state (GS) population due to electron–hole recombination in CsPbI_3_ with the following defects: (a) Cs_I_, (b) I_i_ for hole trapping pathway, (c) I_i_ for electron trapping pathway, and (d) I_v_. The results obtained with the short 1 ps sampling reproduce the standard, long sampling results within 10–20%. The charge recombination time is of the same order in all systems, with and without midgap trap states, confirming that defects are generally benign in lead halide perovskites.

In summary, we have demonstrated that practically useful information on excited-state dynamics in materials can be obtained from short, not fully equilibrated MD trajectories, providing a computational savings of nearly 1 order of magnitude compared to the standard NAMD simulation protocol. Such estimates are particularly important if one needs to screen multiple systems to obtain initial information that can provide leads for further, more thorough analyses. Examples include screening of material stoichiometries and structures, defects in different oxidation states, dopants in various locations, surfaces, grain boundaries, and interfaces. For reliable results, it is essential to obtain a reasonable initial structure. In most cases, systems fluctuate around equilibrium geometries, and the trajectory length is determined by the time scale of such fluctuations. For point defects, dopants, and other local structures, fluctuations proceed on a subpicosecond time scale, and 1–2 ps trajectories are sufficient. In some cases, systems can undergo a structural evolution upon heating, and such a process should be captured. We demonstrate both types of scenarios with the point defects in MHPs, which are anharmonic materials exhibiting complex structural dynamics. The 2 ps nonequilibrium heating trajectories starting from optimized structures are sufficient in the studied examples.

Defects, dopants, nonstoichiometric compositions, grain boundaries, and interfaces present in modern materials give rise to anharmonic motions. Short MD trajectories are advantageous to the harmonic approximation around the optimized geometry and can lead to qualitatively different conclusions, as demonstrated with the Cs_I_ defect in CsPbI_3_, since structures can change upon heating and other perturbations, e.g., strain. Sometimes, very slow motions, such as acoustic modes, glassy dynamics at grain boundaries and interfaces, or defect migration, are essential. The processes require much longer trajectories that can be generated with ML force fields. The 10–20% error created by an order of magnitude shortening of the simulation time is acceptable, given the variety of approximations involved in the NAMD simulations and uncertainties in experimental data on material structure, composition, and time scales. The practical NAMD approach tested here offers substantial computational savings, significantly accelerating screening and rapid exploration and characterization of new materials.

## Supporting Information Available

Comparison of the properties of the systems under investigation obtained with the short and long trajectories (PDF)
