# Supplementary material for: Efficient Modeling of Quantum Dynamics of Charge Carriers in Materials Using Short Nonequilibrium Molecular Dynamics
Source: J Phys Chem Lett. 2023 Sep 8;14(37):8289–95. doi: 10.1021/acs.jpclett.3c02187 (PMC10518862; doi:10.1021/acs.jpclett.3c02187)
Supplement: Supplementary file 1 — jz3c02187_si_001.pdf [file jz3c02187_si_001.pdf]

Supporting Information for:

Efficient Modeling of Quantum Dynamics of Charge Carriers in Materials Using Short Non-Equilibrium Molecular Dynamics

Bipeng Wang,[1] Yifan Wu,[2] Dongyu Liu,[3] Andrey S. Vasenko,[3,4] David Casanova,[4,5]

Oleg V. Prezhdo[1,2,*]

[1]*Department of Chemical Engineering, University of Southern California, Los Angeles, CA 90089, USA*

[2]*Department of Chemistry, University of Southern California, Los Angeles, CA 90089, USA*

[3]*HSE University, 101000 Moscow, Russia*

[4]*Donostia International Physics Center (DIPC), 20018 San Sebastián-Donostia, Euskadi, Spain*

[5]*IKERBASQUE, Basque Foundation for Science, 48009 Bilbao, Euskadi, Spain*

[*] Corresponding author. Email: prezhdo@usc.edu

Table S1. Canonically Averaged (Mean) Energy Gap, Root Mean Square (RMS) Gap, Average Absolute NAC, Root Mean Square NAC, Recombination Time, and Pure-Dephasing Time for the Short (1 ps NVT) and Long (7 ps NVE) Calculation for the Cs_I Defect.

<table>
<tr><td rowspan="3">Cs_I</td><td colspan="6">Gap (eV)</td><td colspan="6">Abs NAC (meV)</td></tr>
<tr><td colspan="2">VBM-Trap</td><td colspan="2">VBM-CBM</td><td colspan="2">Trap-CBM</td><td colspan="2">VBM-Trap</td><td colspan="2">VBM-CBM</td><td colspan="2">Trap-CBM</td></tr>
<tr><td>Mean</td><td>RMS</td><td>Mean</td><td>RMS</td><td>Mean</td><td>RMS</td><td>Mean</td><td>RMS</td><td>Mean</td><td>RMS</td><td>Mean</td><td>RMS</td></tr>
<tr><td>1ps NVT</td><td>1.24</td><td>1.30</td><td>2.06</td><td>2.06</td><td>0.82</td><td>0.88</td><td>0.29</td><td>0.35</td><td>0.30</td><td>0.41</td><td>1.48</td><td>1.89</td></tr>
<tr><td>7ps NVE</td><td>1.34</td><td>1.35</td><td>2.04</td><td>2.04</td><td>0.70</td><td>0.73</td><td>0.24</td><td>0.30</td><td>0.27</td><td>0.37</td><td>0.90</td><td>1.45</td></tr>
<tr><td rowspan="2"></td><td colspan="6" rowspan="2">Recombination Time (ns)</td><td colspan="6">Pure-Dephasing Time (fs)</td></tr>
<tr><td colspan="2">VBM-Trap</td><td colspan="2">VBM-CBM</td><td colspan="2">Trap-CBM</td></tr>
<tr><td>1ps NVT</td><td colspan="6">54</td><td colspan="2">2.50</td><td colspan="2">7.83</td><td colspan="2">2.50</td></tr>
<tr><td>7ps NVE</td><td colspan="6">41</td><td colspan="2">3.76</td><td colspan="2">5.96</td><td colspan="2">3.40</td></tr>
</table>

Table S2. Canonically Averaged (Mean) Energy Gap, Root Mean Square (RMS) Gap, Average Absolute NAC, Root Mean Square NAC, Recombination Time, and Pure-Dephasing Time for the Short (1 ps NVT) and Long (7 ps NVE) Calculation for the I_i Defect.

I_i	Gap (eV)						Abs NAC (meV)					
	VBM-Trap		VBM-CBM		Trap-CBM		VBM-Trap		VBM-CBM		Trap-CBM	
	Mean	RMS	Mean	RMS	Mean	RMS	Mean	RMS	Mean	RMS	Mean	RMS
1ps NVT	0.24	0.29	1.96	1.96	1.72	1.73	0.53	0.69	0.48	0.63	0.25	0.30
7ps NVE	0.33	0.37	1.98	1.98	1.65	1.66	0.94	1.47	0.43	0.55	0.21	0.26

	Recombination Time (ns)	Pure-Dephasing Time (fs)		
		VBM-Trap	VBM-CBM	Trap-CBM
1ps NVT e-trap/h-trap	26/46	4.61	6.59	3.69
7ps NVE e-trap/h-trap	33/52	3.75	6.08	3.46

Table S3. Canonically Averaged (Mean) Energy Gap, Root Mean Square (RMS) Gap, Average Absolute NAC, Root Mean Square NAC, Recombination Time, and Pure-Dephasing Time for the Short (1 ps NVT) and Long (7 ps NVE) Calculation for the I_v Defect.

I_v	Gap (eV)		Abs NAC (meV)	
	VBM-CBM		VBM-CBM	
	Mean	RMS	Mean	RMS
1ps NVT	1.69	1.70	0.70	0.82
7ps NVE	1.69	1.69	0.46	0.55
	Recombination Time (ns)		Pure-Dephasing Time (fs)	
1ps NVT	31		4.75	
7ps NVE	25		5.17	

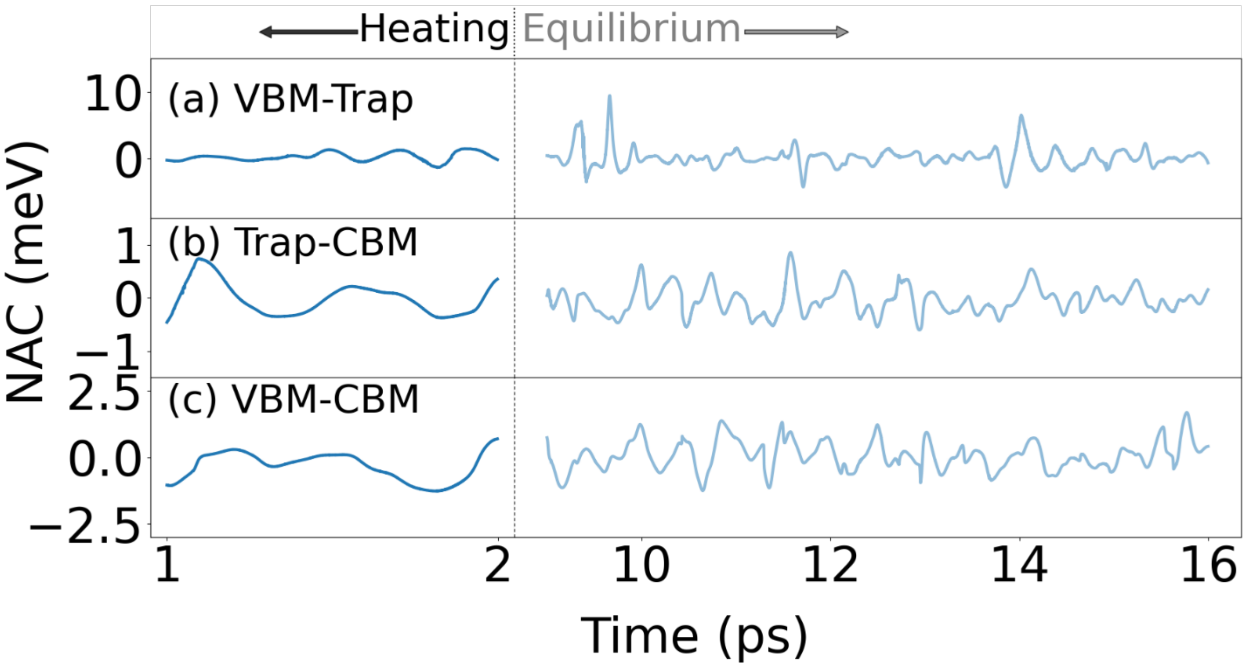

Figure S1. NAC of $CsPbI_3$ with the I_i defect for the **(a)** VBM-trap, **(b)** trap-CBM and **(c)** VBM-CBM transitions. Shown are only the regions selected to test the electron-hole recombination, grey areas in Figure 2. The heating and equilibrium processes are divided by the dotted line. The 1 ps heating region provides a reasonable representation of the NAC in the 7 ps equilibrium region.

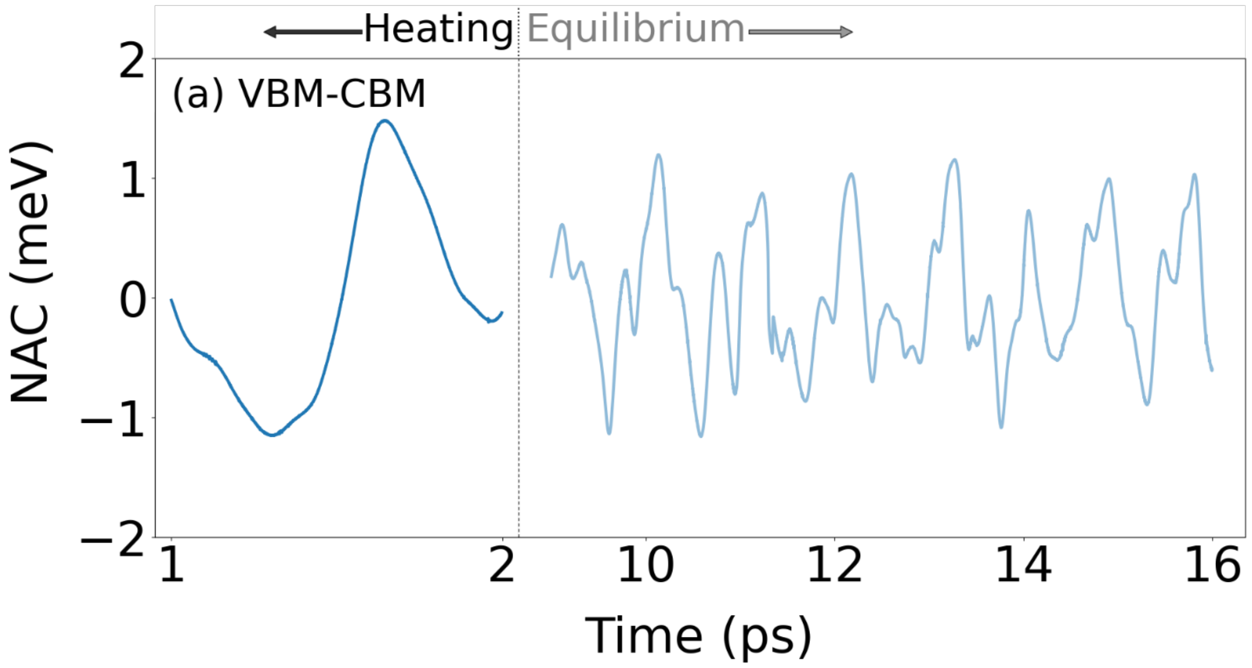

Figure S2. NAC of $CsPbI_3$ with the I_v defect for the VBM-CBM transition. Shown are only the regions selected to test the electron-hole recombination, grey areas in Figure 2. The heating and equilibrium processes are divided by the dotted line. The 1 ps heating region provides a reasonable representation of the NAC in the 7 ps equilibrium region.
